# Genetic variants in tamoxifen metabolism and early treatment discontinuation among premenopausal breast cancer patients

**DOI:** 10.1007/s10549-025-07719-1

**Published:** 2025-05-14

**Authors:** Kirsten M. Woolpert, Thomas P. Ahern, James W. Baurley, Maret L. Maliniak, Per Damkier, Anders Kjærsgaard, Lindsay J. Collin, Stephen Hamilton-Dutoit, Trine Tramm, Bent Ejlertsen, Henrik T. Sørensen, Timothy L. Lash, Deirdre P. Cronin-Fenton

**Affiliations:** 1 Department of Clinical Epidemiology, Department of Clinical Medicine, Aarhus University and Aarhus University Hospital, Aarhus, Denmark; 2 Department of Surgery, The Robert Larner, M.D. College of Medicine at the University of Vermont, Burlington, VT USA; 3 BioRealm, Walnut, CA USA; 4 Department of Epidemiology, Rollins School of Public Health, Emory University, Atlanta, GA USA; 5 Department of Clinical Pharmacology, Odense University Hospital, Odense, Denmark; 6 Department of Clinical Research, University of Southern Denmark, Odense, Denmark; 7 Department of Population Health Sciences, Huntsman Cancer Institute, University of Utah, Salt Lake City, Utah USA; 8 Department of Pathology, Aarhus University Hospital, Aarhus, Denmark; 9 Danish Breast Cancer Group, Department of Oncology, Copenhagen University Hospital, Rigshospitalet, Denmark; 10 Department of Oncology, Copenhagen University Hospital, Rigshospitalet, Denmark; 11 Department of Clinical Medicine, Faculty of Health and Medical Sciences, University of Copenhagen, Copenhagen, Denmark

**Keywords:** Tamoxifen metabolism, Treatment discontinuation, Premenopausal breast cancer, Bayesian methods, CYP2D6, Metabolic pathways

## Abstract

**Purpose:**

Premenopausal, estrogen receptor (ER)-positive breast cancer patients should receive tamoxifen for at least 5 years, but many prematurely discontinue. Activation, transport, and deactivation of tamoxifen and its metabolites are controlled by proteins encoded by genes with functional variations. We examined the impact of genetic polymorphisms in the tamoxifen pathway on early treatment discontinuation.

**Methods:**

We included premenopausal women diagnosed with ER-positive breast cancer (2002–2011) in Denmark who initiated tamoxifen. We genotyped 26 genetic variants in 15 enzymes involved in tamoxifen metabolism. Early discontinuation was defined as tamoxifen use for < 5 years. We estimated individual and combined effects of genetic variants using a Bayesian pathway approach. We report Bayes Factors (BF), wherein values > 1 indicate support of an effect of the genetic pathway on discontinuation (compared with no effect).

**Results:**

Among 3,729 patients, 536 (14%) discontinued tamoxifen within 5 years. Genetic variants involved in tamoxifen activation impacted early discontinuation (BF = 7.5), in a manner driven almost entirely by *CYP2D6* activity (BF = 22.6). Several variants in *CYP2D6* and transporter genes synergistically increased the hazard of early discontinuation (e.g., CYP2D6*2 and *ABCC2;* BF = 138).

**Conclusions:**

Variants in enzymes responsible for activating tamoxifen metabolites—particularly within CYP2D6—influence early tamoxifen discontinuation. *CYP2D6* variants synergistically interact with transporter gene variants, namely *ABCC2*, to further raise the risk of discontinuation.

## Introduction

**Fig. 1:**
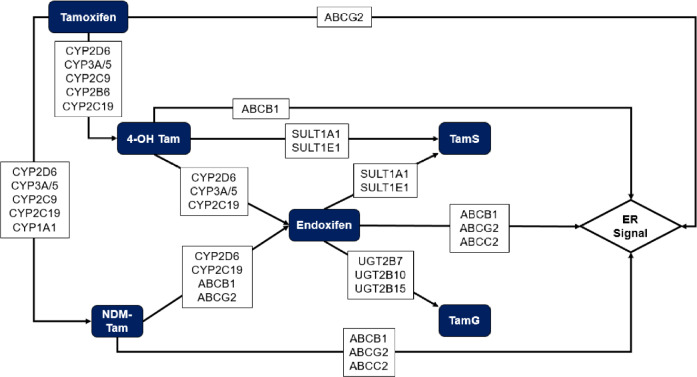
Tamoxifen metabolic pathway. Abbreviations: 4-OH TAM, 4-hydroxy tamoxifen; NDM-TAM, N-desmethyl tamoxifen; TamG, tamoxifen glucuronide; TamS, tamoxifen sulfate; ER, estrogen receptor, Blue boxes represent the tamoxifen metabolites. White boxes represent the polymorphic genes encoding the enzymes responsible for the transitions between the metabolites and the estrogen receptor signal (denoted by the arrows). Figure adapted from Baurley et al., [27] and Ahern et al., [13].

Approximately 70% of premenopausal breast cancer patients have estrogen receptor (ER)-positive (ER +) tumors [1]. Guideline care for these women includes at least 5 years of tamoxifen, which roughly halves recurrence risk [2]. The administered form of tamoxifen is metabolized into more active metabolites with higher ER affinity (*i.e.*, 4-hydroxy tamoxifen [4-OH TAM], N-desmethyl-tamoxifen [NDM-TAM], and 4-hydroxy-N-desmethyl-tamoxifen [endoxifen]) in phase I metabolism [3–5]. Transporter proteins facilitate the movement of these metabolites across cell membranes [6–8]. Phase II metabolic reactions produce excretable metabolites (tamoxifen glucuronides and sulfates) with little or no pharmacologic activity that are rapidly eliminated [9–11]. Several enzymes responsible for these reactions are encoded by genes with functional polymorphisms, which may interact and yield varying concentrations of active metabolites, thereby potentially modifying drug effectiveness (Fig. 1) [12]. We previously found that polymorphic genes encoding enzymes in phase I metabolism were associated with clinical response to adjuvant tamoxifen, as measured by recurrence [13].

Women treated with tamoxifen experience different clinical outcomes, and have varying tolerability to the drug. Our study showed that about 22% of premenopausal breast cancer patients in Denmark prematurely discontinued endocrine therapy [14]. Discontinuation was associated with higher risk of recurrence compared with completion of treatment (HR = 1.67, 95% CI: 1.25, 2.14) [14]. Lower cancer stage, no chemotherapy, greater comorbidity burden, and lower social support are associated with poorer adherence to endocrine therapy over time [15, 16]. Understanding the myriad of factors influencing adherence to endocrine therapy is critical for designing interventions to prevent recurrence and optimize survival.

Genetic differences in tamoxifen metabolism may also modify treatment adherence. Tamoxifen has many adverse effects, including depression and hot flashes, which impact treatment adherence [17]. Higher serum endoxifen levels are associated with more severe adverse effects [18–20]. Most prior research has focused on variants in *CYP2D6* genes [3]. Reduced *CYP2D6* activity from such variants is associated with lower endoxifen concentrations, potentially reducing tamoxifen effectiveness, while increasing the likelihood of treatment adherence [3, 18, 21]. Few studies have specifically investigated tamoxifen discontinuation—a potential indicator of clinical response—as an endpoint. A Swedish study found that *CYP2D6* ultrarapid metabolizers were more likely to prematurely discontinue tamoxifen [22]. Compared with normal metabolizers, poor and ultrarapid metabolizers had excess breast cancer mortality [22]. This finding emphasizes the need for further research on gene-induced modifications associated with tamoxifen discontinuation. Given the complexity of tamoxifen metabolism, multiple enzymes and transporters contribute to its activation and clearance, making it challenging to isolate the impact of individual genes. Single-enzyme analyses may oversimplify the pharmacokinetic processes involved, but conventional epidemiologic approaches often struggle to capture the many interactions within metabolic pathways.

Here, we used a Bayesian pathway modeling approach to investigate whether genetic variant interactions within key enzymes and transporters known to be involved in tamoxifen metabolism—including *CYP2D6, CYP3A4, CYP2C19*, and efflux and uptake transporters—might influence treatment discontinuation rates among a cohort of premenopausal women with breast cancer.

## Methods

### Study population

The Predictors of Breast Cancer Recurrence cohort (ProBe CaRe) includes all premenopausal women diagnosed with stage I–III breast cancer from 2002 to 2011 in Denmark who were registered with the Danish Breast Cancer Group (DBCG) [23]. This study involved ER + ProBe CaRe participants who initiated adjuvant tamoxifen and had genetic data previously collected from available tumor tissue blocks (86% of the entire cohort) [23]. Approval for this study was granted by the Regional Ethics Committee of Central Denmark (journal number 1–10-72–22-13), the Danish Breast Cancer Group, and the Danish Data Protection Agency (Aarhus University number 2016–051-000001, #458) and adhered to the General Data Protection Regulation.

### Genetic polymorphisms in tamoxifen metabolism

We considered functional polymorphisms in key phase I, phase II, and transporter genes (Supplementary Figure S1 A-C) with minor allele frequency (MAF) ≥ 5% that were genotyped in an earlier analysis [13]. In brief, formalin-fixed, paraffin-embedded tumor tissue blocks were collected from treating hospitals and used to obtain DNA for genotyping. More information on this is outlined in our previous publication [13]. For each polymorphism in the 15 enzymes, individuals were characterized as having two, one, or no functional alleles. In the case of missing genetic data for individual variants, we imputed 50 datasets based on patient and tumor characteristics, as well as observed genotypes at other loci (Supplementary Figure S2) [13]. We then aggregated all datasets into one complete dataset for analysis. Several variants (rs1048943, rs28371706, rs1042157, rs1801030, rs10248420, and rs2231164) were excluded from the analyses because of meaningful departure from expected frequencies under Hardy–Weinberg equilibrium or because of poor minor allele amplification [13, 24].

### Tamoxifen discontinuation

Patients with breast cancer in Denmark are invited to follow DBCG protocols, which for ER + women diagnosed during the time period of our cohort involved biannual follow-up visits with the treating oncologist [25]. At these visits, women reported whether they were still taking tamoxifen. On average, women continuing treatment received a 6-month resupply of tamoxifen directly from their treating oncologist, and not at a pharmacy like in other healthcare settings. We created supply diaries using this information from the DBCG for each patient, starting from the first registration of tamoxifen (*i.e.,* initiation) and spanning 5 years after diagnosis. The diaries allowed for carry-over of up to 6 months of medication if tamoxifen was resupplied before the previous supply was exhausted. Supply diaries were censored at death, recurrence, first registration of an aromatase inhibitor (indicating transition to menopause), emigration, or 5 years after diagnosis. Using this information from the diaries, we defined early discontinuation as having ≥ 182 consecutive days (i.e., 6 months) with no available tamoxifen. We then considered the date of discontinuation as the 182nd consecutive day of no available tamoxifen. Time to discontinuation was calculated as the time between tamoxifen initiation and the date of discontinuation.

### Statistical analysis

We calculated frequencies and proportions of the included patients according to clinical and demographic characteristics. We fit conventional Cox proportional hazards models to estimate the hazard ratios (HRs) and 95% confidence intervals (95% CIs) associating individual genetic variants with early tamoxifen discontinuation. To account for multiple comparisons, we also applied empirical Bayes shrinkage without setting a prior [26]. In these models, the genetic variant was treated numerically, so that the HR represented the increase in tamoxifen discontinuation hazard for every unit increase in the number of minor alleles. The covariates included in the models were age at diagnosis (years), UICC pathologic stage (I/II/III), surgery type (mastectomy/lumpectomy), receipt of radiotherapy (yes/no), receipt of chemotherapy (yes/no), histologic grade (I/II/III/not graded), HER2 status (negative/positive/unknown or not measured), cohabitation status (cohabiting/living alone), and employment status at diagnosis (employed/not working).

To evaluate the likelihood of early tamoxifen discontinuation according to the complex network of metabolic and transporter genetic variants, we used the Algorithm for Learning Pathway Structures (ALPS) [27, 28]. As previously demonstrated, ALPS characterizes the effects of individual genes, gene combinations, and pathway concepts on a given outcome by modeling multiplicative interactions between genetic variants [13, 27, 28]. Steps for running ALPS have been described elsewhere [27, 28] and are summarized below:

1. *Encode the prior*: We codified the interrelationships among the 26 genetic variants in 15 enzymes involved in tamoxifen metabolism into a prior pathway (Fig. 1).
2. *Calculate prior probabilities*: We ran ALPS for 3 million iterations without any genetic data (*i.e.*, with only the prior pathway) to obtain prior probabilities, which were calculated by dividing the number of times in which a particular pathway feature (*i.e.,* tree) was visited by the total number of iterations.
3. *Calculate posterior probabilities*: We then ran ALPS for 1 million iterations, this time including complete data on genetic variants and time to discontinuation. ALPS finds a random spot in the prior pathway to begin a Markov chain Monte Carlo (MCMC) search. At each iteration, it proposes a change to the structure of the examined tree and computes the marginal likelihood of the posterior tree, which is accepted or rejected with Metropolis–Hastings probability before proceeding to the next iteration [28].
4. *Summarize results*: We analyzed the trees accepted during the Metropolis–Hastings step, summarized information regarding the identified posterior trees, and calculated posterior probabilities. We then calculated Bayes Factors (BF) as the quotient of the posterior and prior odds for (A) individual variants, (B) pathway concepts (*e.g.,* “phase I metabolism,” Supplementary Figure S2), and (C) top identified trees (BF ≥ 10 and posterior probability ≥ 0.01). A BF > 1 indicates that the data support the hypothesis that a given feature affects discontinuation (rather than having no effect). The magnitude of support for this hypothesis increases with the magnitude of the BF. We also calculated the log hazard of early discontinuation by using the partial likelihoods calculated in Step 3, as previously described (Supplementary Methods) [27]. Further information is outlined in the online supplement.

All analyses were conducted using R version 4.0 (Vienna, Austria). Code for the implementation of ALPS is available at: https://github.com/tpahern/ALPS-Bayesian-Pathway-Analysis.

## Results

The final cohort of patients who initiated adjuvant tamoxifen and had available genetic information included 3,729 women (Table 1; flowchart for study inclusion in Supplementary Figure S3). The median age at diagnosis was 46 years (interquartile range: 42–49); most patients had stage II disease (55%) and were treated with chemotherapy before initiating tamoxifen (92%). We identified 536 women (14%) who prematurely discontinued tamoxifen, after accounting for 1,767 women (47%) who initiated aromatase inhibitors during follow-up, and were thus censored on the date of their switch.

**Table 1:** Descriptive characteristics of 3,729 estrogen receptor-positive breast cancer patients who initiated adjuvant tamoxifen and had available genetic data in the Predictors of Breast Cancer Recurrence (ProBe CaRe) cohort

	Included patients, n (%)
Total	3,729 (100)
Early discontinuation of tamoxifen	536 (14)
Age at diagnosis	
< 40	570 (15)
40–49	2,341 (63)
50–55	818 (22)
Stage at diagnosis^a^	
Stage I	957 (26)
Stage II	2,034 (55)
Stage III	738 (20)
Positive lymph nodes at diagnosis	
0	1,381 (37)
1–3	1,646 (44)
4–9	482 (13)
10 +	220 (5.9)
Histological grade	
I	795 (21)
II	1,924 (52)
III	758 (20)
Missing/Not graded	252 (6.7)
Tumor size^a^	
< 2 cm	2,139 (57)
2– < 5 cm	1,468 (39)
> = 5 cm	122 (3.3)
HER2 status	
Negative	2,351 (63)
Positive	493 (13)
Unknown/not measured	885 (24)
Treated with chemotherapy	3,382 (91)
Treated with radiation	2,023 (54)
Surgery type	
Mastectomy	1,662 (45)
Lumpectomy	2,067 (55)
Recurrence in first ten years	489 (13)
Death in first ten years (any cause)	512 (12)
Received aromatase inhibitors^b^	1,767 (47)

^a^Missing stage and tumor size were multiply imputed using available patient characteristics. This has been previously described and impacted less than 1% of the study population [15]

^b^Patients were censored on the date of a switch to aromatase inhibitors

**Fig. 2:**
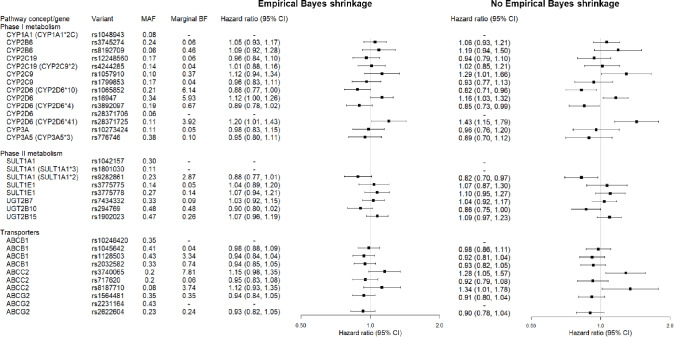
Algorithm for Learning Pathway Structure (ALPS) Bayes Factors and multivariable adjusted hazard ratios (Cox regression models) for the relationship between individual genetic variants and time to tamoxifen discontinuation. Abbreviations: ALPS, Algorithm for Learning Pathway Structure; MAF, minor allele frequency; CI, confidence interval; CYP, cytochrome P450; SULT, sulfotransferase; UGT, uridine 5’-diphosphoglucuronosyltransferase; ABC, ATP-binding cassette. **a**. Multivariable models represent the hazard of discontinuation for each unit-increase in the number of minor alleles in the selected gene. **b**. Models adjusted for age at diagnosis, stage at diagnosis, surgery type, histologic grade, receipt of radiotherapy, receipt of chemotherapy, HER2 status, cohabitation status, and employment status at diagnosis

Three of the four investigated variants in *CYP2D6* were identified by ALPS to have some evidence of an association with early tamoxifen discontinuation (CYP2D6*10 [rs1065852] BF: 6.1; rs16947 BF: 5.9; CYP2D6*41 [rs28371725] BF: 3.9) (Fig. 2). In conventional analyses, we also observed some signals of an effect on discontinuation, for example, the CYP2D6*10 variant was associated with a decreased risk of early discontinuation (HR: 0.88, 95% CI: 0.77, 1.00). The fourth *CYP2D6* variant investigated, CYP2D6*4 (rs3892097), was not identified by ALPS to be associated with discontinuation (BF: 0.67). Most genes in phase II metabolism were identified in neither ALPS nor conventional analyses to be associated with early discontinuation, except for SULT1A1*2 (rs9282861) (BF: 2.9; HR: 0.88, 95% CI: 0.77, 1.01). Variants in the *ABCC2* transporter gene were also identified in ALPS. The highest BF of 7.8 was observed for *ABCC2* (rs3740065), which corresponded in conventional analyses to an increased hazard of early discontinuation, based on comparison of an increasing number of minor alleles versus wild-type homozygotes (HR: 1.15, 95% CI: 0.98, 1.35). ALPS diagnostics can be found in Supplementary Table S1 and results for the relationship between individual genetic variants modeled as factor variables and early discontinuation in Supplementary Tables S2 & S3.

When combining the variants to investigate the effects of summary pathway concepts, we observed that variants in phase I metabolism influenced early discontinuation (BF = 7.5) (Table 2). The involvement of variants in *CYP2D6* genes in early tamoxifen discontinuation was associated with discontinuation (BF = 22.6), whereas other phase I genes, such as *CYP3A* (BF = 0.1), were not associated with discontinuation. Variants in the synthesis of the metabolites produced in phase I metabolism, including 4-OH-TAM, NDM-TAM, and endoxifen, also showed some evidence of an association with early discontinuation (BF = 8.6, 8.6, and 5.6, respectively). Phase II metabolism did not appear to be associated with discontinuation (BF = 0.4), but the transporters involved in drug efflux did show some evidence of an association (BF = 3.3).

**Table 2:** Algorithm for Learning Pathway Structure (ALPS) Bayes Factors for the relation between tamoxifen metabolic pathway concepts and early discontinuation of tamoxifen among 3,729 premenopausal breast cancer patients

Pathway Concept	ALPS BFs
Enzyme function	
Phase I metabolism	7.5
Phase II metabolism	0.4
Transporters	3.3
Tamoxifen metabolites	
4-Hydroxy tamoxifen	8.6
N-Desmethyl tamoxifen	8.6
4-Hydroxy-N-desmethyl tamoxifen	5.6
Tamoxifen sulfate	0.5
Tamoxifen glucuronide	0.3
*CYP2D6* activity	22.6
*CYP3A* activity	0.1

Abbreviations: ALPS, Algorithm for Learning Pathway Structures; BFs, Bayes Factors

BFs greater than 1 indicate that the data support an effect on discontinuation

^a^Some of the genetic variants included in each pathway concept were excluded from posterior analysis because they showed meaningful departure from the expected genotype distribution under Hardy–Weinberg assumptions or had poor amplification of the minor alleles

**Fig. 3:**
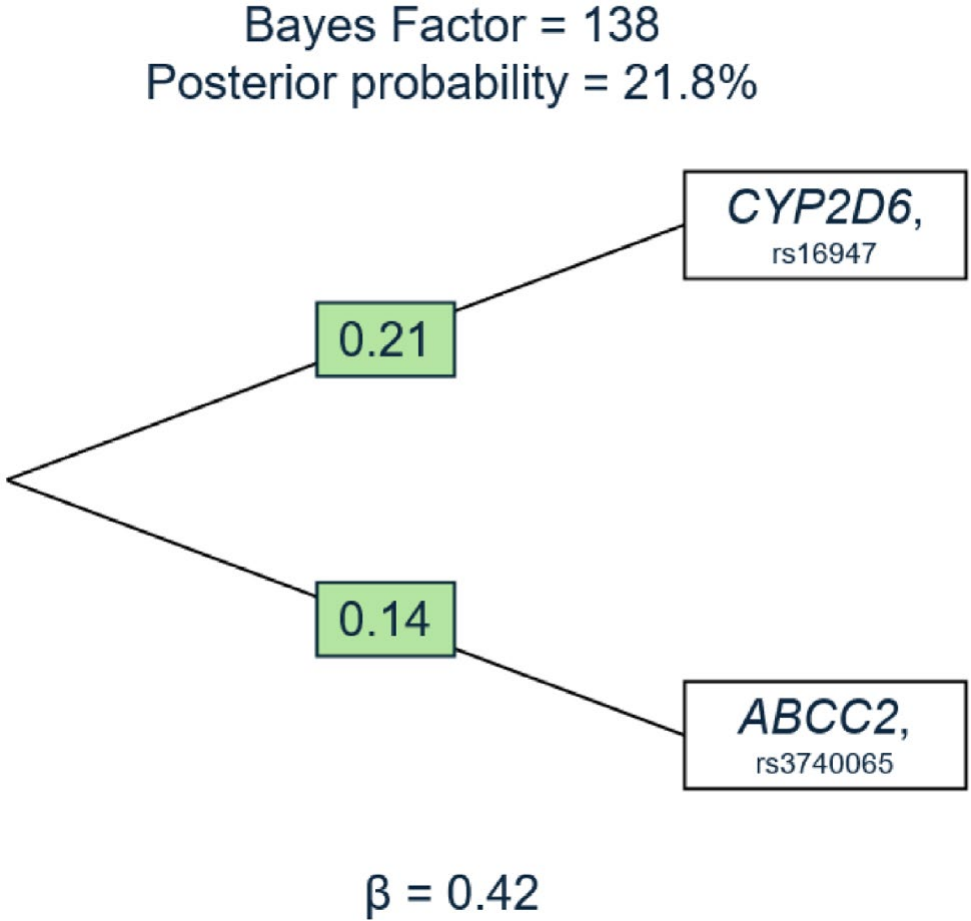
Top tree-structure identified in the Algorithm for Learning Pathway Structure (ALPS) among 3,729 premenopausal breast cancer patients representing the association between the interaction between *CYP2D6* and *ABCC2* and early discontinuation of tamoxifen. Abbreviations: ALPS, Algorithm for Learning Pathway Structure; CYP, cytochrome P450; ABC, ATP-binding cassette. **a**. The green boxes show the individual theta (θ) values. These represent the quantification of the interaction between the two genes. **b**. The beta (β) represents the overall pathway effect of both genes on early discontinuation of tamoxifen

ALPS also identified specific combinations of genetic variants (*i.e.,* trees) that are associated with time to discontinuation. Eight tree structures had a BF ≥ 10 and a posterior probability ≥ 0.01, six of which involved combinations of variants in *CYP2D6* and *ABC* transporter genes (Supplementary Tables S4 & S5). The other two identified trees included combinations of variants in *CYP2D6* and *SULT1A1*. As an example, we show the calculations for the top identified tree, which involved variants in *CYP2D6* (rs16947) and *ABCC2* (rs3740065) and had a BF of 138 (Fig. 3). The ALPS-derived hazard ratios are displayed in Table 3. Women with two variants in each gene had a risk of premature tamoxifen discontinuation four-fold that of women with no variants in both genes. In the full ProBe CaRe cohort, 775 women (21%) had at least one variant in each of these two genes.

**Table 3:** Hazard ratios of early tamoxifen discontinuation among 3,729 premenopausal breast cancer patients derived from Algorithm for Learning Pathway Structure (ALPS) analytic output for the combinations of genotypes in the top ALPS-identified tree (*ABCC2* and *CYP2D6*)

		*CYP2D6*, rs16947 Minor allele: A		
		T / T	T / A	A / A
*ABCC2*, rs3740065 Minor allele: T	A / A	1 (ref)	1.09	1.19
	A / T	1.06	1.52	2.18
	T / T	1.12	2.12	4.00

Abbreviations: ALPS, Algorithm for Learning Pathway Structure; CYP, cytochrome P450; ABC, ATP-binding cassette

## Discussion

Our findings suggest that phase I metabolism—which involves the formulation of 4-OH-TAM, NDM-TAM, and endoxifen—is associated with early discontinuation of tamoxifen. These findings were driven almost entirely by variants in *CYP2D6*. We observed no association between phase II genetic variants and treatment discontinuation but found some evidence of a role of transporters. We additionally observed potential gene interactions between variants in *CYP2D6* and several *ABC* transporter genes that were associated with early tamoxifen discontinuation.

The combination of all investigated *CYP2D6* genes had the strongest association with early discontinuation, substantiating the hypothesis that CYP2D6 function is associated with tamoxifen adherence. Investigations regarding variants in *CYP2D6* and clinical response to tamoxifen have a long and somewhat controversial history in breast cancer research. Some studies have shown that variants in *CYP2D6* increase the risk of breast cancer recurrence [29–32] and overall mortality [22, 29, 30, 33, 34], whereas others have found no association [35–39]. Additionally, several studies have investigated concomitant use of CYP2D6-inhibiting medications and tamoxifen response, and also have yielded inconsistent findings [37, 40–43]. In 2018, the Clinical Pharmacogenetics Implementation Consortium published guidelines recommending *CYP2D6* genotype-guided tamoxifen prescribing [44], but the ESMO clinical practice guidelines in 2019 stated that *CYP2D6* polymorphisms should not be used as a decision aid [45]. Associations between *CYP2D6* and recurrence and mortality might be mediated, at least partly, by rates of tamoxifen discontinuation.

The observed interaction between *CYP2D6* and *ABC* variants on early discontinuation might suggest synergism between polymorphisms in *CYP2D6* (rs16947) and *ABCC2* (rs3740065). This interaction was previously associated with breast cancer recurrence. Kiyotani et al. observed an increasing hazard of recurrence as the number of risk alleles in both *CYP2D6* and *ABCC2* (rs3740065) increased [32]. Although this study was compromised by immortal time bias and low statistical power for analyses of gene–gene interactions, it is interesting that relatively common polymorphisms in these two genes have been identified to predict both discontinuation and recurrence in separate populations. Limited literature has described the roles of transporters in tamoxifen metabolism, thus highlighting an important area for future research. The link between CYP2D6 function and active metabolite levels is well established [46], but the level of active metabolites being transported across cells might be hypothesized to be further associated with early treatment discontinuation.

No data on tamoxifen-associated adverse effects were available in this study, but prior research suggests that genetic variants influencing tamoxifen metabolism may contribute to side effects, which in turn impact adherence and recurrence risk. Higher concentrations of active tamoxifen metabolites improve clinical outcomes but are also linked to greater side effect burden, which may lead to early discontinuation [47–53]. For example, high 4-OH-TAM was associated with vaginal dryness, which was also a predictor of early treatment discontinuation (HR: 3.72, 95% CI: 1.49, 9.72) [54]. CYP2D6 ultrarapid metabolizers have shown increased use of medications for nausea, anxiety, and hot flashes, along with higher early discontinuation rates (HR: 2.06, 95% CI: 1.11, 3.82) [22]. Although we did not have data on adverse effects, the observed associations in the current study may reflect differences in metabolite levels that influence both tolerability and early discontinuation. However, this remains a hypothesis, as we could not directly assess whether adverse effects mediated the relationship between genetic variation and discontinuation in our study.

This study had several limitations. First, not all genes involved in the metabolism of tamoxifen were included. As variants with a MAF ≤ 5% were not genotyped, our findings may be missing other contributors to early discontinuation. This study also only considered genetic pathways of tamoxifen metabolism, and no other pathways that could relate to adverse effects and early discontinuation. A key limitation is that genetic data were derived from formalin-fixed paraffin-embedded tumor tissue rather than blood, preventing us from fully assessing CYP2D6 metabolizer status, which is typically based on many polymorphisms. Since we did not genotype all these variants, our results refer only to individual polymorphisms and their interactions, without providing information on overall enzyme activity. We also conducted this study among patients with available tumor tissue, which resulted in the exclusion of ~ 15% of patients who would have been eligible for the study had genetic data been available. The collection of tumor samples is routinely archived in the Danish Pathology Data Bank [55], but was not possible for the entire ProBe CaRe cohort. Additionally, the generalizability of our findings is limited. The ProBe CaRe cohort comprises primarily white women of European descent. Some variants identified in this study have markedly different MAFs in different populations. Finally, misclassification might have existed in our definition of tamoxifen discontinuation, which assumed that the women took the medication as dispensed. However, our definition was conservative, allowing for dosage carry-over and requiring a full 6-month gap in coverage before considering a woman to have discontinued therapy. This aspect would be expected to have biased our results toward the null.

In this comprehensive pharmacogenetic study among 3,729 premenopausal women with breast cancer, genetic variants involved in phase I metabolism, particularly among *CYP2D6* genes, were associated with early tamoxifen discontinuation. Variants in *CYP2D6* may synergistically interact with transporter genes in their relationship with discontinuation. These findings contribute to ongoing discussion on how to improve overall adherence to tamoxifen therapy, and potentially, improve long-term patient outcomes.

## Data Availability

The compilation and analysis of data in this study were conducted within the secure servers of Statistics Denmark and are not publicly available in accordance with Danish privacy laws. Procedures for accessing the data and a detailed study protocol can be made available by contacting the corresponding author.
